# Tuberculosis in people newly diagnosed with HIV at a large HIV care and treatment center in Northwest Cameroon: Burden, comparative screening and diagnostic yields, and patient outcomes

**DOI:** 10.1371/journal.pone.0199634

**Published:** 2018-06-26

**Authors:** Eyongetah Tabenyang Mbu, Florian Sauter, Alexander Zoufaly, Barend M. de C. Bronsvoort, Kenton L. Morgan, Jürgen Noeske, Jean-Louis Foe Abena, Melissa S. Sander

**Affiliations:** 1 Approved Treatment Center for HIV/AIDS, Bamenda Regional Hospital, Bamenda, Cameroon; 2 The Roslin Institute, University of Edinburgh, Edinburgh, United Kingdom; 3 Institute of Veterinary Science, University of Liverpool, Leahurst, United Kingdom; 4 Senior Consultant, Yaounde, Cameroon; 5 National TB Programme, Yaounde, Cameroon; 6 Tuberculosis Reference Laboratory Bamenda, Bamenda, Cameroon; Central University of Tamil Nadu, INDIA

## Abstract

**Background:**

Diagnosis of tuberculosis in people living with HIV is challenging due to non-specific clinical presentations and inadequately sensitive diagnostic tests. The WHO recommends screening using a clinical algorithm followed by rapid diagnosis using the Xpert MTB/RIF assay, and more information is needed to evaluate these recommendations in different settings.

**Methods:**

From August 2012 to September 2013, consecutive adults newly diagnosed with HIV in Bamenda, Cameroon, were screened for TB regardless of symptoms by smear microscopy and culture; the Xpert MTB/RIF assay was performed retrospectively. Time to treatment and patient outcomes were obtained from routine registers.

**Results:**

Among 1,149 people enrolled, 940 (82%) produced sputum for lab testing; of these, 68% were women, the median age was 35 years (IQR, 28–42 years), the median CD4 count was 291cells/μL (IQR, 116–496 cells/μL), and 86% had one or more of current cough, fever, night sweats, or weight loss. In total, 131 people (14%, 95% CI, 12–16%) had sputum culture-positive TB. The WHO symptom screening algorithm had a sensitivity of 92% (95%CI, 86–96%) and specificity of 15% (95%CI, 12–17%) in this population. Compared to TB culture, the sensitivity of direct smear microscopy was 25% (95% CI, 18–34%), and the sensitivity of Xpert was 68% (95% CI, 58–76); the sensitivity of both was higher for people reporting more symptoms. Only one of 69 people with smear-negative/culture-positive TB was started on TB treatment prior to culture positivity. Of 71 people with bacteriologically-confirmed TB and known outcome after 6 months, 13 (17%) had died, including 11 people with smear-negative TB and 6 people with both smear and Xpert-negative TB.

**Conclusions:**

Use of the most sensitive rapid diagnostic test available is critical in people newly diagnosed with HIV in this setting to maximize the detection of bacteriologically-confirmed TB. However, this intervention is not sufficient alone and should be combined with more comprehensive clinical diagnosis of TB to improve outcomes.

## Introduction

Detecting tuberculosis in people with the disease is often a significant challenge since screening algorithms are typically non-specific and the currently available rapid diagnostic tools are inadequately sensitive. These challenges are compounded in people living with HIV, who present with more highly variable clinical manifestations of TB than people without HIV.[1–3] As a result, the mortality rate among people with HIV and TB co-infection is high, and many of these people die without ever being diagnosed with TB.[4]

To improve TB case finding among people living with HIV, in 2011 the World Health Organization (WHO) recommended regular screening using a clinical algorithm.[5,6] In a high TB prevalence setting, a person living with HIV presenting with current cough, fever, night sweats and/or weight loss is to be evaluated further to either identify TB or make another diagnosis and initiate isoniazid preventive therapy for TB. In most settings, smear microscopy is the only widely available laboratory tool for diagnosis of TB, and although it is rapid and inexpensive, it has poor sensitivity, particularly in people living with HIV.[7–9] Since 2010, the WHO has recommended replacing smear microscopy with the Xpert MTB/RIF assay,[10] which is rapid and has better sensitivity but is also significantly more expensive than microscopy. The performance of the recommended WHO symptom screen combined with the Xpert MTB/RIF assay testing has been assessed in various settings.[11–13]. The development of optimal local algorithms for TB diagnosis depends on setting-specific factors, including disease burden, resource availability, and public health priorities.[14,15]

Cameroon has a high estimated incidence of TB/HIV (70 per 100,000), a modest estimated TB case detection rate (55%), and a low coverage of testing with WHO-recommended rapid diagnostics for TB (Xpert MTB/RIF assay) as an initial diagnostic test (<10%).[16] The performance of the current WHO recommendations for symptom screening and Xpert MTB/RIF testing among people living with HIV have not yet been evaluated in this setting. The aim of this study was to assess the burden of TB among people newly diagnosed with HIV at a large testing and treatment center in Cameroon and to compare the performance of the WHO-recommended clinical screening algorithm and TB diagnostic methods in this population.

## Methods

### Study setting and methods

This study was conducted at the Treatment Center for HIV/AIDS at the Bamenda Regional Hospital and at the Tuberculosis Reference Laboratory Bamenda. The Treatment Center served as the largest of 18 such centers in the Northwest Region of Cameroon at the time of the study; currently there are a total of 81 HIV care and treatment centers serving this region. This center reported ~1,200 newly diagnosed HIV cases each year in 2012 and 2013, which represented approximately one third of all HIV cases diagnosed in the Northwest geographical region during this time. The study was planned to include 780 participants with TB lab testing results, from whom there would be an estimated 69 TB cases based on a TB prevalence of 8.5% from a meta-analysis including people attending HIV care and treatment centers.[1] The Tuberculosis Reference Laboratory Bamenda is an accredited facility (SANAS accredited medical laboratory, No. M0593). The laboratory works with the National TB Program and serves four regions of the country. This study was approved by the National Ethics Committee of Cameroon.

Consecutive adults with newly-diagnosed HIV infection presenting at the HIV testing and ART treatment center at the Bamenda Regional Hospital from August 6, 2012 to September 3, 2013 were screened for eligibility and invited to participate. Participants were recruited regardless of the presence or absence of symptoms or clinical suspicion of TB. People were included if they had a first diagnosis of HIV within the past month, were ≥18 years of age and provided written informed consent; people were excluded if they were currently on TB treatment. After enrollment, the participants completed a standardized interview, underwent a physical exam to aid in WHO staging, provided blood for CD4 testing, and were instructed on how to produce two sputum specimens for TB lab testing, one on the spot and one early in the morning.

People diagnosed with bacteriologically-confirmed TB were followed up in the routine service. The time to initiation of treatment and vital status at 6 months were recorded from the patient records.

### Laboratory methods

For each specimen, a smear was prepared directly from sputum and examined for acid-fast bacilli by fluorescence microscopy, then the sputum was processed using the N-acetyl-L-cysteine–NaOH (NALC-NaOH) method.[17] From the re-suspended pellet, a concentrated smear was prepared and examined for acid-fast bacilli by fluorescence microscopy, 0.5mL was cultured on mycobacterial growth indicator tubes (MGIT, BD Diagnostic Systems, Sparks, MD, USA) using the BACTEC MGIT 960 system, and ~0.1mL was cultured on each of two slopes of Löwenstein-Jensen media. Cultures were read by lab technicians who did not have knowledge of microscopy results. Cultures positive for acid-fast bacilli were tested by MPT64 antigen detection (Standard Diagnostics, Korea), and those that were positive were identified as *M*. *tuberculosis* complex. The remainder of the processed sputum was stored at -20°C because the Xpert assay was not available in our setting at the time of the study. The remainder from the morning sputum specimen from those with culture-confirmed TB was tested retrospectively using the Xpert MTB/RIF assay (Cepheid, Sunnyvale, USA). Retrospective Xpert testing was only possible for 108 of the 131 specimens from people with culture-positive TB because some specimens were inadvertently discarded.

### Definitions

People with one or more sputum culture positive for *M*. *tuberculosis* complex were defined as having tuberculosis, while those with at least one negative culture and no positive cultures were defined as not having tuberculosis. Sputum smears with a grade of scanty, 1+, 2+ or 3+ were classified as smear-positive; for those providing two sputum specimens, the higher result was used to classify by smear-grade and as smear-positive or smear-negative. Participants were followed up by the routine HIV and TB services. Laboratory and TB treatment registers were reviewed for patients diagnosed with TB to determine vital status from clinical outcome (cured on treatment, died before or during treatment, lost to follow-up before or during treatment, transferred out or referred to another site for treatment).

### Data analysis

Data was entered into an EpiData (www.epidata.dk) database from the study files and laboratory registers and validated by double entry and comparison. Participants were characterized using simple descriptive statistics. The sensitivity of microscopy and Xpert MTB/RIF assay were calculated and stratified by number of symptoms reported, WHO stage, CD4 cell count and patient outcome. Proportions were compared using the χ^2^ or Fisher’s exact test as appropriate. EpiData was used for analysis. The STROBE recommendations were followed for reporting these data.[18]

## Results

**Fig 1:**
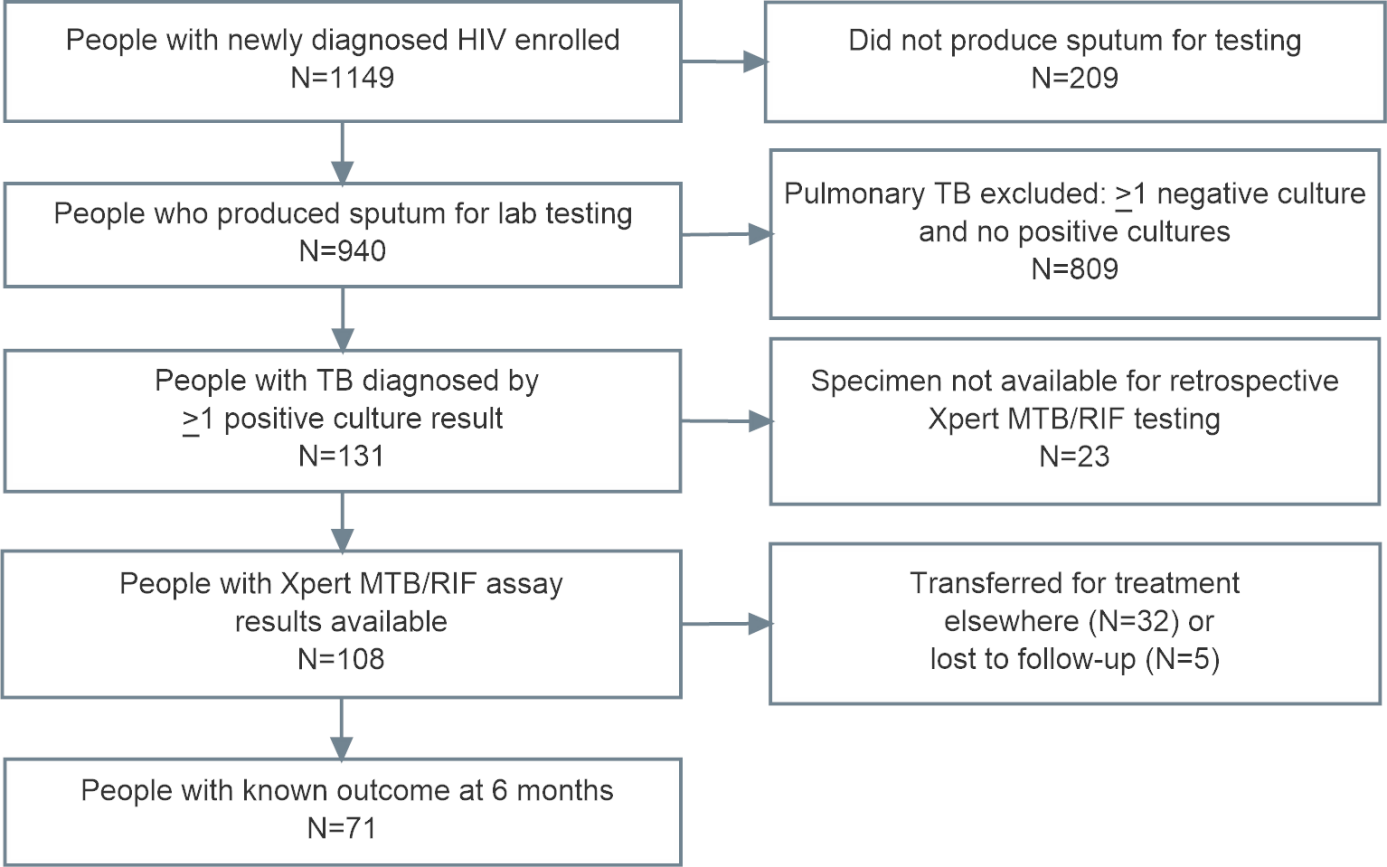
Flow diagram showing the study population.

Of 1,149 people who were enrolled and screened for TB, 940 (82%) produced at least one sputum specimen for laboratory testing (902 of these produced two sputum specimens), and 131 had at least one sputum culture positive for *M*. *tuberculosis* complex (Fig 1). Of those with culture-positive TB, the Xpert MTB/RIF assay was performed retrospectively on the remaining sputum for 108 (82%). Among these, 71 people (66%) had a known vital status at six months.

Among those tested for TB (Table 1), 68% were women, the median age was 35 years (IQR, 28–42 years), and the median CD4 count was 291cells/μL (IQR, 116-496cells/mL). In this population, 86% of participants had a positive WHO symptom screen, including 48% with current cough, 23% with fever, 42% with night sweats, and 63% with weight loss. These characteristics were similar among the 209 people who did not produce sputum for testing, of whom 70% were women, with median age of 32 years (IQR, 27–41 years), median CD4 count of 289 cells/μL (IQR, 108–512 cells/μL) and 86% with a positive WHO symptom screen of current cough, fever, night sweats and/or weight loss.

**Table 1:** Characteristics of 940 people newly diagnosed with HIV for whom TB culture results were available, according to whether TB was diagnosed.

Characteristic		All participants		No bacteriologically-confirmed TB		Bacteriologically-confirmed TB	
		(n = 940)		(n = 809)		(n = 131)	
**Age**							
	Age, median (IQR), years	35	(28–42)	34	(28–42)	36	(29–43)
**Sex**							
	Female, % (n)	68	(641)	70	(569)	55	(72)
	Male, % (n)	32	(299)	30	(240)	45	(59)
**WHO stage** (n = 821)*							
	1–2, % (n)	66	(539)	70	(490)	40	(49)
	3–4, % (n)	34	(282)	30	(209)	60	(73)
**CD4 count (cells/μL)**							
	CD4, median (IQR)	291	(116–496)	315	(139–511)	143	(42–276)
**Symptoms**							
	Positive WHO screen, % (n)	86	(812)	85	(691)	92	(121)

* 119 people did not have a WHO stage recorded. IQR: interquartile range

Overall, the prevalence of sputum culture-positive TB in this population was 13.9% (95% CI, 11.9–16.3%). As in Table 2, the prevalence was higher among men than women (20% vs. 11%, p<0.001). The prevalence varied greatly when stratified by CD4 count, from 7% for people with a CD4>350 cells/uL to 26% among those with CD4<100 cells/uL. For people reporting any one of cough, fever, night sweats or weight loss, the prevalence was 15% (95% CI, 13–18%), while for those reporting none of these symptoms, it was 8% (95% CI, 4–14%). Among people reporting any cough, the TB prevalence was 20%, and this was similar for those reporting a cough for more or less than 2 weeks (22% vs. 18%, respectively).

**Table 2:** Tuberculosis prevalence and number needed to test (NNT) to identify one TB case among 940 people newly diagnosed with HIV, stratified by characteristics.

Characteristic		All	Number of TB cases	TB prevalence, % (95% CI)		p-value	Number needed to test, NNT
**All patients**	** **	**940**	**131**	**14**	**(12–16)**	** **	7
Age	≤35 years	510	63	12	(10–16)	0.13	8
	>35 years	430	68	16	(13–20)		7
Sex	Female	641	72	11	(9–14)	**<0.001**	9
	Male	299	59	20	(16–25)	** **	5
CD4 count, cells/μL	>350	387	25	7	(4–9)	**<0.001**	14
	200–349	212	26	12	(9–17)	** **	8
	100–199	128	24	19	(13–26)	** **	5
	<100	213	56	26	(21–33)	** **	4
WHO stage	1 or 2	539	49	9	(7–12)	**<0.001**	11
	3 or 4	282	73	26	(21–31)	** **	4
Number of symptoms*	No symptoms	128	10	8	(4–14)	**<0.001**	13
	Any 1 symptom	812	121	15	(13–18)	** **	7
	Any 2 symptoms	523	104	20	(17–24)	** **	5
	Any 3 symptoms	245	69	28	(23–34)	** **	4
	All 4 symptoms	75	33	44	(33–55)	** **	2
Symptom	Current cough	448	88	20	(16–24)	**<0.001**	5
	No current cough	492	43	9	(7–12)	** **	11
	Cough lasting ≥2 weeks	228	49	22	(17–27)	0.34	5
	Cough lasting <2 weeks	220	39	18	(13–23)		6
	Current fever	220	54	25	(19–31)	**<0.001**	4
	No current fever	720	77	11	(9–13)	** **	9
	Night sweats, any	395	81	21	(17–25)	**<0.001**	5
	No night sweats	545	50	9	(7–12)	** **	11
	Weight loss	592	104	18	(15–21)	**<0.001**	6
	No weight loss	348	27	8	(5–11)	** **	13

*Symptoms including current cough, fever, night sweats, weight loss

In these people with newly-diagnosed HIV infection, the WHO screening algorithm (current cough, fever, night sweats and/or weight loss) had a sensitivity of 92% (95% CI, 86–96%), specificity of 15% (95% CI, 12–17%), positive predictive value of 15% (95% CI, 14–17%), and negative predictive value of 92% (95% CI, 86–96%) to detect TB when compared with the culture as the reference standard.

Of the 131 people with culture-confirmed TB, the sensitivity of direct smear microscopy was 24% (95% CI, 18–32%), the sensitivity of concentrated smear microscopy was 35% (95% CI, 28–44%), the sensitivity of solid culture was 81% (95% CI, 73–87%) and the sensitivity of liquid culture was 98% (95% CI, 94–99%). The final contamination rate on solid culture was 4.3% and on liquid culture was 3.3%, and there were no specimens with all cultures contaminated. Of the 131 people with culture-confirmed TB, 85 (65%) had two specimens culture positive, 38 (29%) had one of two specimens culture positive, and 8 (6%) had only one specimen submitted and culture positive (S1 Table).

For retrospective testing of Xpert on a single specimen, the sensitivity of direct and concentrated smear microscopy for the 108 specimens included was similar to that for the 131 people with culture confirmed TB (25% vs. 24% and 36% vs. 35%, respectively). The sensitivity of Xpert compared to culture-positive TB was 68% (95% CI, 58–76%); the Xpert sensitivity was 97% among the 39 smear-positive specimens and 51% among the 69 smear-negative specimens. Of the 73 Xpert positive specimens, 20 (27%) had an Xpert grade of very low, 27 (37%) low, 19 (26%) medium, and 7 (10%) high positive, (S1 Table).

As shown in Table 3, for those people reporting all four symptoms, the sensitivity of microscopy and Xpert was higher, 61% and 86% respectively, than in those people reporting no symptoms, 17% and 50%, respectively. Smear microscopy also had a higher sensitivity among those classified as WHO stage 3 or 4 as compared to WHO stage 1 or 2 (50% vs. 18%, p = 0.001). However, while diagnostic testing sensitivity was higher for people with indicators of more severe disease, neither microscopy nor Xpert had higher sensitivity to detect those with TB who had died within 6 months of diagnosis as opposed to those who were still alive.

**Table 3:** The sensitivities of microscopy and Xpert MTB/RIF assay among 108 people with culture-positive TB, stratified by number of symptoms reported, WHO stage, CD4 count and vital status at 6 months.

	Microscopy (conc.)*			Xpert MTB/RIF		
	%	(95% CI)	p- value	%	(95% CI)	p-value
**Total (n = 108)**	**36**	**(27–46)**	** **	**68**	**(58–76)**	
**Number of symptoms reported**	** **	** **	**0.03**	** **	** **	0.09
No symptoms (n = 6)	17	(0–64)		50	(12–88)	
1 symptom (n = 16)	19	(4–46)	** **	69	(41–89)	
2 symptoms (n = 27)	30	(14–50)	** **	52	(32–71)	
3 symptoms (n = 36)	32	(17–51)	** **	68	(49–83)	
4 symptoms (n = 28)	61	(41–79)	** **	86	(67–96)	
**WHO stage** *(n = 100)*			**0.001**			0.13
1 or 2 (n = 40)	18	(7–33)		58	(41–73)	
3 or 4 (n = 60)	50	(37–63)	** **	73	(60–84)	
**CD4 count (cells/uL)**	** **	** **	0.1	** **	** **	0.4
≥200 (n = 42)	26	(14–42)		62	(46–76)	
<200 (n = 66)	42	(30–55)		71	(59–81)	
**Patient vital status at 6 months** *(n = 71*)†	** **	** **	0.06	** **	** **	0.52
Alive (n = 58)	45	(32–58)		67	(54–79)	
Died (n = 13)	15	(2–45)		54	(26–80)	

*Microscopy results are for smears prepared from pellets after processing sputum for culture (the sensitivity of direct smear microscopy was 25%, 95% CI 18–34%).

†37 people did not have known vital status at 6 months because they had either been referred for treatment at another site or were lost to follow-up.

At the end of six months, 58 people (82%) with known outcome were alive and cured of TB, while 13 people (18%) had died, including 4 who were smear-negative and died before being diagnosed with culture-positive TB. Of those who were known to have died, 10 (73%) were smear-negative and 6 (46%) were both smear- and Xpert-negative. The remaining 37 people had unknown vital status at 6 months, including 27 who were referred for TB treatment elsewhere, 5 who were transferred to another site after starting treatment, 3 (2 smear-positive and 1 smear-negative) who were lost to follow-up on treatment and 2 (both smear-negative) who were lost to follow-up before starting treatment.

Of the 67 people who started TB treatment, the median time from the microscopy result to treatment start was 17 days (IQR, 2–27 days). The time to start treatment for those with smear-positive TB was 2 days (IQR, 1–3 days), while those with smear-negative TB started treatment after 26 days (IQR, 20–32 days).

In this cohort, only one of the people with smear-negative TB was started on TB treatment prior to the result of culture-positive TB; the other 68 people with smear-negative TB who were started on TB treatment were all started as bacteriologically-confirmed TB cases following the positive culture result.

## Discussion

Among people newly diagnosed with HIV at a large HIV care and treatment center in Cameroon, 86% reported one or more symptoms of TB, and 14% (131/940) had culture-confirmed pulmonary TB. The WHO symptom screen (current cough, fever, night sweats, weight loss) was 92% sensitive and 15% specific to detect bacteriologically-confirmed TB in this population. Direct smear microscopy detected 25% of people with culture-positive TB, concentrated smear microscopy detected 36%, and retrospective testing with the Xpert MTB/RIF assay detected 68%. Both smear microscopy and the Xpert MTB/RIF assay had higher sensitivity to detect TB among people reporting more symptoms, but neither had good sensitivity to detect TB among those people who died during follow-up. These findings suggest that while intensified screening and more sensitive diagnostics can add significant value to improving TB case finding, these approaches should be combined with more intensive clinical follow-up of people newly diagnosed with HIV in order to improve outcomes.

The proportion of people notified with clinically-diagnosed TB (as opposed to bacteriologically-confirmed TB) among all pulmonary TB cases in Cameroon is 26%, while it is 33% in the African region and 43% globally.[16] This appears to reflect a more conservative approach to the clinical diagnosis of TB in Cameroon than in other settings. This is particularly notable since the only TB diagnostic tool available in nearly the entire country is direct smear microscopy, which detected only 25% of culture-confirmed TB cases among people living with HIV in this study.

The prevalence of bacteriologically-confirmed TB among people newly diagnosed with HIV in this population was 14% (95%CI, 12–16%), which falls at the higher end of the range reported from a meta-analysis of TB prevalence among people newly diagnosed with HIV at antiretroviral and medical clinics (median 8.6%, range 3.6–24.7%).[1] In our study, the median CD4 cell count of participants was relatively low (291 cells/uL) and 34% were classified as WHO stage 3 or 4, which indicates that many of those newly diagnosed with HIV had been infected for some time before their HIV diagnosis and were at a more advanced stage of HIV disease. Since the time of the study, the approach to HIV treatment in Cameroon has changed from initiation on antiretrovirals with advancing disease (CD4<350 cells/uL or WHO stage 3/4) to the Test and Treat strategy. As testing increases and HIV infection is detected earlier in the course of the disease, it is anticipated that the burden of TB among those newly diagnosed with HIV will decrease[19] and clinical presentations of TB will shift towards less advanced disease.[20]

The sensitivity of the Xpert MTB/RIF assay compared to TB culture in this population (68%, 95% CI, 58–76) is somewhat lower than what has been reported previously for people living with HIV in a meta-analysis (79%, 95%CI 70–86%)[21]. It is closer to the lower Xpert sensitivity reported when compared to culture on two specimens rather than a single specimen as the reference standard (76%, 95% CI, 66–84%)[22], which is expected since we used two specimens for the culture reference standard in this work. While the sensitivity of the Xpert MTB/RIF assay was 43% higher than direct microscopy and 32% higher than concentrated microscopy among the patients in this study, Xpert testing still missed 32% of those with culture-positive pulmonary TB. Better performing diagnostic tests are urgently needed to continue to improve TB control efforts and improve patient outcomes.

People reporting more symptoms and those with higher WHO stage (3 or 4) were more likely to have smear-positive TB than those reporting fewer symptoms or having less advanced disease by WHO stage. These findings are in agreement with others who have reported increased sputum mycobacterial load among people with TB having highly advanced HIV disease, as reflected in higher Xpert MTB/RIF sensitivity[7,23,24] and increasing sensitivity of smear microscopy as CD4 cell count dropped below 150 cells/uL[25].

Of the 131 people with culture-confirmed TB, 88 (67%) reported current cough while 33 (25%) did not report cough but reported one or more of fever, night sweats or weight loss. This highlights the importance of collecting sputum from all people reporting any symptoms of TB, even those who do not report having cough and may need additional encouragement to produce sputum. An additional 10 people (8% of all culture-positive TB cases) reported none of the 4 symptoms, including 1 with smear-positive TB. Similar amounts of asymptomatic, culture-positive TB have been reported in other settings. [15,26,27]

This study had several limitations. Participants were enrolled only from a single site, and although the Bamenda care and treatment center is one of the largest in the country, the generalizability of the findings needs to be further assessed. While we performed a total of six sputum cultures per person (one liquid and two solid cultures for each of two specimens), which helps to provide a strong estimate of the prevalence of pulmonary TB in this population, we did not perform culture on non-sputum specimens. The overall prevalence of TB is therefore underestimated since a significant proportion of TB in people living with HIV is extrapulmonary. In addition, our assessment of patient vital status at six months was limited due to high rates of transfer (30%) and loss to follow up (5%).

These findings highlight the need for the use of better diagnostic tools and algorithms to diagnose TB among people living with HIV, as well as emphasis on careful follow-up of people evaluated for TB in this setting. More work is needed to evaluate how to effectively follow-up people with TB who have a negative lab test to ensure they are diagnosed and linked to treatment as quickly as possible. These actions are necessary to facilitate faster detection of TB cases and to improve patient outcomes, particularly among people living with HIV who have smear and/or Xpert-negative TB.

## Supporting information

S1 Table Sensitivity of TB detection by type of diagnostic method and specimen among 131 people newly diagnosed with HIV with culture-confirmed TB (Table A). Comparison of results by Xpert grade, microscopy result, and type of microscopy for 108 people newly diagnosed with HIV with culture-confirmed TB and an Xpert MTB/RIF assay result (Table B).
(PDF)
