# Supplementary material for: Tuberculosis in people newly diagnosed with HIV at a large HIV care and treatment center in Northwest Cameroon: Burden, comparative screening and diagnostic yields, and patient outcomes
Source: PLoS One. 2018 Jun 26;13(6):e0199634. doi: 10.1371/journal.pone.0199634 (PMC6019259; doi:10.1371/journal.pone.0199634)
Supplement: S1 Table — Sensitivity of TB detection by type of diagnostic method and specimen among 131 people newly diagnosed with HIV with culture-confirmed TB (Table A). Comparison of results by Xpert grade, microscopy result, and type of microscopy for 108 people newly diagnosed with HIV with culture-confirmed TB and an Xpert MTB/RIF assay result (Table B). (PDF) [file pone.0199634.s001.pdf]

Table A. Sensitivity by type of diagnostic method and specimen among 131 people newly diagnosed with HIV with culture-confirmed TB

	1st sputum specimen n=131		2nd sputum specimen* n=123		Combined- 1st+2nd specimens n=131	
**Diagnostic method**	%	95% CI	%	95% CI	%	95% CI
Direct smear	18.3	(12.6-25.8)	22.8	(16.2-30.9)	24.4	(17.9-32.4)
Concentrated smear	29.8	(22.6-38.1)	34.1	(26.4-42.9)	35.1	(27.5-43.6)
Solid culture	69.5	(61.1-76.7)	65.0	(56.3-72.9)	80.9	(73.3-86.7)
Liquid culture	78.6	(70.8-84.8)	83.7	(76.2-89.2)	97.7	(93.5-99.2)
Either culture	84.0	(76.7-89.3)	86.2	(79.0-91.2)	Reference	

**8 people (6%) had only one sputum specimen submitted.*

Table B. Comparison of TB diagnostic results by Xpert grade, microscopy result, and type of microscopy for 108 people newly diagnosed with HIV with an Xpert result and culture-confirmed TB

	**Microscopy results**			
	Direct smear		Concentrated smear	
**Xpert MTB/RIF result**	Positive	Negative	Positive	Negative
MTB not detected (n=35)	0	35	1	34
MTB detected, very low (n=20)	2	18	3	17
MTB detected, low (n=27)	2	25	10	17
MTB detected, medium (n=19)	16	3	18	1
MTB detected, high (n=7)	7	0	7	0
Total (n=108)	27	81	39	69
